# Use of sirolimus as an adjuvant therapy for kidney transplant recipients with high-risk cutaneous squamous cell carcinomas: a prospective non-randomized controlled study

**DOI:** 10.1590/2175-8239-JBN-2023-0013en

**Published:** 2023-08-11

**Authors:** Marina Rezende de Fázio, Marina Pontello Cristelli, Jane Tomimori, Carlos Eiji Koga, Marília Marufuji Ogawa, Giovanni Tani Beneventi, Helio Tedesco-Silva, José Medina-Pestana

**Affiliations:** 1 Universidade Federal de São Paulo, Hospital do Rim, Divisão de Nefrologia, São Paulo, SP, Brazil.; 2 Universidade Federal de São Paulo, Divisão de Dermatologia, São Paulo, SP, Brazil.

**Keywords:** Kidney Transplant Recipients, Cutaneous Squamous Cell Carcinoma, Immunosuppression, Sirolimus, Receptores de Transplante Renal, Carcinoma Espinocelular Cutâneo, Imunossupressão, Sirolimo

## Abstract

**Introduction:**

Previous research demonstrated benefits of late conversion to mTOR inhibitors against cutaneous squamous cell carcinomas (cSCC) in kidney transplant recipients (KTR), despite of poor tolerability. This study investigated whether stepwise conversion to sirolimus monotherapy without an attack dose modified the course of disease with improved tolerability.

**Methods:**

This prospective exploratory study included non-sensitized KTR with more than 12-months post-transplant, on continuous use of calcineurin inhibitors (CNI)-based therapy, and with poor-prognosis cSCC lesions. Incidence densities of high-risk cSCC over 3-years after conversion to sirolimus-monotherapy were compared to a non-randomized group with high-risk cSCC but unsuitable/not willing for conversion.

**Results:**

Forty-four patients were included (83% male, mean age 60 ± 9.7years, 62% with skin type II, mean time after transplantation 9 ± 5.7years). There were 25 patients converted to SRL and 19 individuals kept on CNI. There was a tendency of decreasing density of incidence of all cSCC in the SRL group and increasing in the CNI group (1.49 to 1.00 lesions/patient-year and 1.74 to 2.08 lesions/patient-year, p = 0.141). The density incidence of moderately differentiated decreased significantly in the SRL group while increasing significantly in the CNI group (0.31 to 0.11 lesions/patient-year and 0.25 to 0.62 lesions/patient-year, p = 0.001). In the SRL group, there were no sirolimus discontinuations, no acute rejection episodes, and no *de novo* DSA formation. Renal function remained stable.

**Conclusions:**

This study suggests that sirolimus monotherapy may be useful as adjuvant therapy of high-risk cSCC in kidney transplant recipients. The conversion strategy used was well tolerated and safe regarding key mid-term transplant outcomes.

## RESUMO

**Introdução:**

Pesquisas anteriores demonstraram benefícios da conversão tardia para inibidores de mTOR contra carcinomas espinocelulares cutâneos (CECs) em receptores de transplante renal (RTR), apesar da baixa tolerabilidade. Este estudo investigou se a conversão gradual para monoterapia com sirolimo sem dose de ataque modificou o curso da doença com melhor tolerabilidade.

**Métodos:**

Esse estudo prospectivo exploratório incluiu RTR não sensibilizados com mais de 12 meses pós-transplante, uso contínuo de terapia imunossupressora baseado em inibidor de calcineurina (CNI) associado a micofenolato de sódio ou azatioprina, com lesões de CECs de mau prognóstico. Comparou-se densidades de incidência de CECs de alto risco durante 3 anos após conversão para monoterapia com sirolimo à um grupo não randomizado com CECs classificados conforme os mesmos critérios de gravidade do grupo sirolimo, mas inadequado/não disposto à conversão.

**Resultados:**

Foram incluídos 44 pacientes (83% homens, idade média 60 ± 9,7 anos, 62% com fototipo de pele II, tempo médio pós-transplante 9 ± 5,7 anos). 25 pacientes foram convertidos para SRL e 19 indivíduos mantidos em CNI. Foi observado tendência de diminuição da densidade de incidência de todos CECs no grupo SRL e de aumento no grupo CNI (1,49 a 1,00 lesões/paciente-ano; 1,74 a 2,08 lesões/paciente-ano; p = 0,141). A densidade de incidência de lesões moderadamente diferenciadas diminuiu significativamente no grupo SRL enquanto aumentou significativamente no grupo CNI (0,31 a 0,11 lesões/paciente-ano; 0,25 a 0,62 lesões/paciente-ano; p = 0,001). No grupo SRL não houve descontinuação do sirolimo, nenhum episódio de rejeição aguda e nenhuma formação de DSA de novo. Função renal permaneceu estável.

**Conclusões:**

Esse estudo sugere que a monoterapia com sirolimo pode ser útil como terapia adjuvante de CECs de alto risco em RTR. A estratégia de conversão usada foi bem tolerada e segura em relação aos principais desfechos do transplante a médio prazo.

## Introduction

Cutaneous squamous cell carcinoma (cSCC) is the most common cancer among kidney transplant recipients, with incidence rates up to 250 times higher than in general population and a higher risk of local recurrence, metastasis, and death^1,2,3,4,5^. In addition to the traditional risk factors such as older age, skin type, chronic sun exposure, smoking, and human papillomavirus (HPV) infection, the type and duration of exposure to immunosuppressive drugs also interfere with the development of cSCC.

The mTOR inhibitors (mTORi) have been shown to have anti-tumor effects by disrupting critical pathways of cell proliferation and angiogenesis, and by limiting the replication of certain oncoviruses such as HPV^1,3,4,6,7^. There were three major randomized controlled trials investigating the effect of conversion from calcineurin inhibitors (CNI) to sirolimus (SRL) in kidney transplant recipients with non-melanoma skin cancer. All these studies demonstrated a significant reduction in the risk of development of new non-melanoma skin lesions, mainly cSCC, after one to two years of follow-up. Importantly, the benefits were not clear among most needed patients with multiple or more aggressive lesions. The high variability of its cytostatic pharmacodynamic effect and the high discontinuation rate due to adverse events, between 23% to 46%, might have been involved^8,9,10,11^. 

Therefore, this exploratory study investigated the disease modifying properties of conversion from CNI to SRL as an adjuvant cytostatic therapy for kidney transplant recipients with cutaneous squamous cell carcinomas designated as aggressive or high-risk cSCC, which has substantially higher rates of recurrence and metastases.

## Methods

This is a single-center, non-randomized, open label, exploratory study designed to investigate the efficacy and safety of a stepwise conversion from CNI- to SRL-based therapy on the incidence and severity of new cSCC in kidney transplant recipients over 36 months of follow-up. The endpoints of interest were compared to a non-randomized control group of patients with high-risk cSCC but unsuitable/not willing for conversion, as detailed below.

This study was conducted in accordance with the Declaration of Helsinki and the International Good Clinical Practices guidelines and was approved by the local ethics committee (CAEE: 40254114.5.0000.5505). All patients provided written informed consent before enrollment.

### Eligibility

Between May 2015 and March 2018, those adult kidney transplant recipients with cSCC lesions considered to be aggressive or with bad evolution despite primary treatment (consisted of surgical clearance) and close follow-up by a dedicated team of dermatologists were invited to participate. After signing the consent form, patients were considered eligible if they had high-risk cSCC as defined below, had more than 12 months after transplantation and, in the past six months, had no treated acute allograft rejection episodes, presented stable kidney function (defined by estimated glomerular filtration rate ≥ 40 mL/min/1.73m^2^ by MDRD-4 equation and no more than 15% variation), and were on continuous use of CNI, prednisone and azathioprine or mycophenolate.

#### Definition of high-risk cSCC

High-risk cSCC was defined as:

1. having more than one active biopsy-confirmed cSCC; and/or
2. at least one lesion located in the scalp, face, or neck, and/or
3. cSCC with moderately or poorly differentiated histology, and/or
4. cSCC with perineural invasion^12,13,14^.

### Screening Visit

At the baseline visit (T0), the eligible patients underwent a clinical and laboratory screening assessment and were considered suitable for intervention if presented pre-transplant panel reactive antibodies <50%, no donor specific antibodies anytime, no history of chronic pulmonary disease, no peripheral lymphedema, no post-transplant glomerulonephritis, estimated glomerular filtration rate ≥ 40 mL/min/1.73m^2^ by MDRD-4 equation, proteinuria < 0,5g, hemoglobin level > 11 g/dL, white blood cell count > 4.000/µL, platelet count > 150.000/µL, fasting triglycerides < 5.65 mmol/L, cholesterol < 3.39 mmol/L, and transaminases < 3 times above upper normal range.

### Stepwise Conversion to Sirolimus

The patients suitable for intervention underwent a stepwise conversion to sirolimus (SRL group). During the first visit, the CNI dose was reduced by 50% and sirolimus was started at 2 mg/day. After achieving a whole blood sirolimus concentration of 10–15 ng/mL, CNI was discontinued. The next visit within one to three weeks, mycophenolate or azathioprine were suspended. Patients were therefore kept on sirolimus plus prednisone (5 mg/day).

#### Non-randomized CNI group

Patients who agreed and were considered eligible to participate, but who were considered unsuitable at the screening visit or refused conversion to SRL were maintained on their CNI-based regimens at the previous target blood concentrations, but submitted to the same follow-up study visits. These patients composed the non-randomized control group (CNI group). The reasons for not converting to sirolimus were detailed in the **Table S1**.

### Follow-up

After conversion to sirolimus, all patients had appointments with a nephrologist with laboratory evaluations in a weekly basis in the first month, biweekly in the second month, monthly between the third and twelfth month, and every 3 months from one year to three years. Patients were evaluated by two dedicated dermatologists at baseline (T0), and at 12 (T1), 24 (T2), and 36 (T3) months. At each visit, a complete physical examination looking for skin lesions was performed, and all the new-suspected lesions were biopsied. The following information was registered: number of new biopsy-proven cSCC lesions; number of cSCC in scalp, face and neck; number of cSCC moderately differentiated; number of cSCC poorly differentiated; number of cSCC with perineural invasion.

The efficacy was evaluated comparing the incidence densities off all the confirmed cSCC observed at each study visit during three years of follow up in the SRL versus the CNI groups. Moreover, we also compared the incidence densities of confirmed cSCC stratified by each high-risk criterion. The safety of the strategy was evaluated by the time course of kidney function, the incidence rates of biopsy-proven acute rejection, and the occurrence of graft loss and recipients’ death. In the SRL group, the occurrence of adverse events, the rates and causes of SRL discontinuation and the development of *de novo* donor specific antibodies after conversion were also analyzed.

### Statistical Analysis

Since this was an exploratory study, no sample size calculation was performed in advance. To obtain the incidence density in the baseline visit, the total number of biopsied-proven cSCC lesions observed during all the historical dermatologic follow-up was divided by the total number of person-years at risk before the inclusion. The time to T0 was calculated from the start of follow-up with the dedicated dermatology team that works regularly in partnership with the transplant center. In each subsequent yearly study visit, the total number of new lesions was divided by the number of person-years at risk in that year. A similar analysis was performed by stratifying the lesions by each severity criterion. The comparative analysis was performed using the Generalized Estimating Equations model with log-link function and Poisson distribution considering the period of exposure, which allows the incorporation of dependence among observations of the same patient. For all statistical tests, a level of 5% significance. Statistical analysis were performed using the STATA 17 statistical software. 

## Results

**Figure 1.**
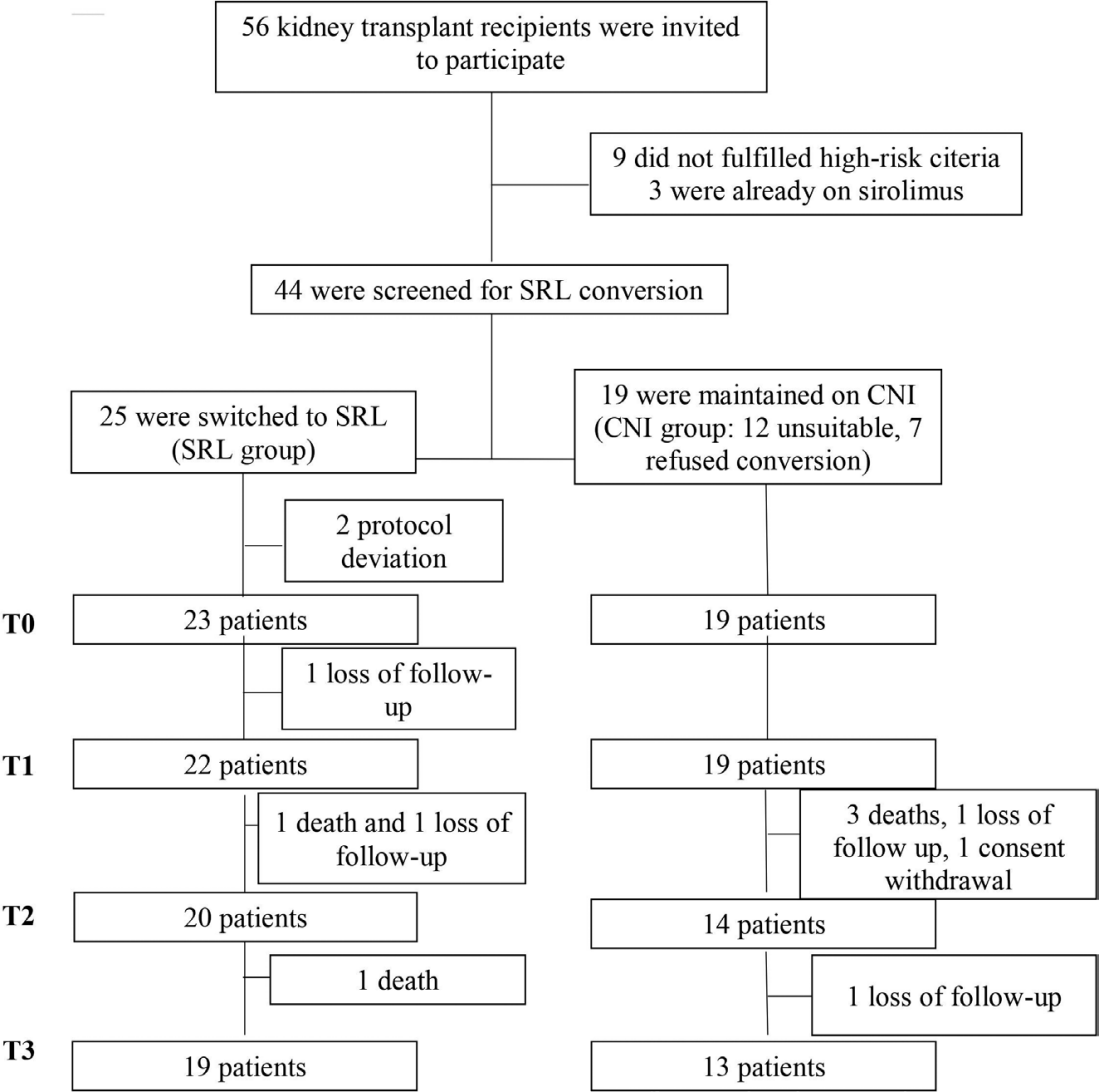
Patient disposition.

Among the 56 patients who signed the consent form, nine did not present high-risk cSCC and other three were already on sirolimus treatment. From the remaining 44 patients, 25 were converted to sirolimus and 19 were kept on CNI-based regimens (12 unsuitable for intervention at the screening visit and seven refusals to conversion; CNI group). During data audit, it was noted that two patients in the SRL group actually did not have high-risk cSCC (protocol deviation). These patients were excluded from the analyses (**Figure 1**).

Baseline demographics were similar between SRL and CNI groups (**Table 1**). Patients were 83% male, with a mean age of 60 ± 9.7 years and 62% with skin type II. 52% of them had received a deceased-donor kidney transplant, only 3 patients were retransplants, and the mean time after transplantation was 9 ± 5.7 years. No induction agent was used in 66% of the patients, and CNI plus prednisone plus azathioprine was the most frequent maintenance regimen in use (73%).

**Table 1:** Baseline characteristics of population of the study at the moment of the screening visit

	SRL group n = 23	CNI group n = 19	p-value
**Demographic and transplant characteristics**		
Male gender, n (%)	20 (87)	15 (79)	0.493
Mean age, years ± SD	61 ± 8	58 ± 10	0.286
Hemodialysis before transplantation, n (%)	21 (91)	17 (89)	0.831
Mean time on dialysis, years ± SD	3.8 ± 3.4	3.2 ± 2.6	0.531
Deceased donor transplant, n (%)	12 (52)	10 (53)	0.949
Mean time after transplant, years ± SD	8.9 ± 4	9.5 ± 6.6	0.718
Retransplant, n (%)	1 (4.4)	2 (10.5)	0.451
Induction therapy, n (%)			
No induction	16 (70)	12 (63)	0.908
Basiliximab	4 (17)	4 (21)	
Thymoglobulin	3 (13)	3 (16)	
Immunosuppressive regimen, n (%)			
Cyclosporine + prednisone + azathioprine	9 (39)	6 (31.5)	0.753
Tacrolimus + prednisone + azathioprine	9 (39)	7 (37.0)	
Tacrolimus + prednisone + mycophenolate	5 (22)	6 (31.5)	
**Baseline dermatological characteristics**		
Skin type, n (%)			
I	1 (4)	0	0.084
II	15 (65)	11(58)	
III	7 (31)	4 (21)	
IV	0	4 (21)
Mean dermatological follow-up before screening, months ± SD	69 ± 58	70 ± 56	0.956
Number of cSCC lesions per patient, n (%)			
1	2 (9)	1 (6)	0.355
2–9	15 (65)	9 (47)	
≥10	6 (26)	9 (47)	
At least one lesion in scalp, face or neck, n (%)	22 (96)	19 (100)	0.383
Moderately differentiated histology, n (%)	19 (83)	14 (74)	0.481
Poorly differentiated histology, n (%)	6 (26)	5 (26)	1.000
Perineural invasion, n (%)	3 (13)	1 (5)	0.381

Regarding dermatological characteristics in the baseline visit **(Table 1)**, 93% of the population of the study had two or more lesions with diagnosis of high-risk cSCC. The three patients who presented a single lesion had other high-risk criteria: one patient in the SRL group had one moderately differentiated facial lesion, another patient in the SRL group had one poorly differentiated facial lesion, and one patient in the CNI group had one poorly differentiated lesion with perineural invasion. Nineth-five percent of the patients in SRL group and all the patients in the CNI group had at least one cSCC lesion was located on the scalp, face or/and neck. Moderately differentiated lesions were present in 82% of the patients in the SRL group and in 73% of patients in CNI group. Poorly differentiated lesions were present in 26% of patients of both groups. Three patients in the SRL group and one patient in the CNI group and presented cSCC with perineural invasion.

### Efficacy

The mean time of historical dermatological follow-up before inclusion in the study was 69 ± 58 months in the SRL group versus 70 ± 56 months in the CNI group (p = 0.956). At baseline (T0), the incidence density of cSCC was 1.49 lesions/patient-year in the SRL group versus 1.74 lesions/patient-year in the CNI group (p = 0.949).

**Figure 2.**
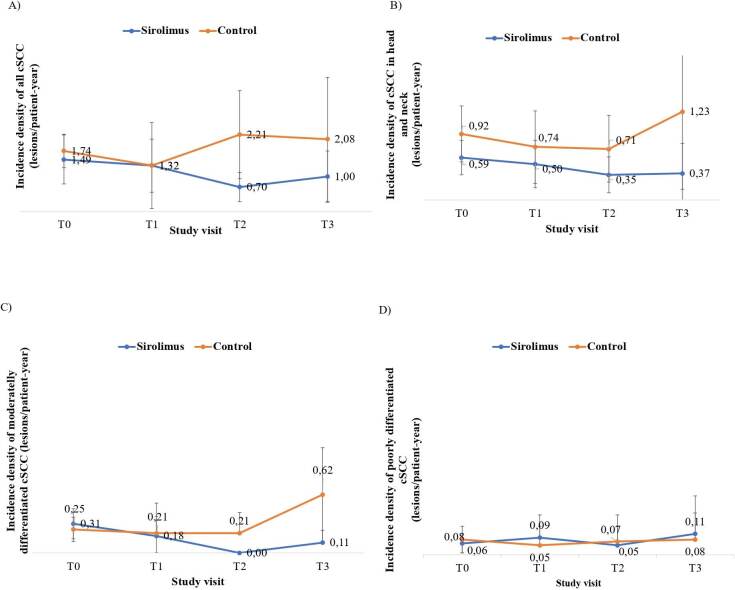
Estimates of incidence density and respective 95% confidence interval of number of (A) all cSCC, (B) cSCC in scalp, head and neck, (C) cSCC moderately differentiated, and (D) cSCC poorly differentiated over time, stratified by sirolimus (blue) or control (orange) study groups.

When analyzing the behavior of all cSCC lesions as a collective, especially from visit T2 on, there was a trend of decreasing incidence densities in the SRL group and a trend of increasing incidence densities in the CNI group over time (p = 0.141; **Figure 2A**).

When stratified by each one of the high-risk criteria, a trend of decreasing incidence density in favor of the sirolimus group was observed in scalp, head and neck lesions over time (**Figure 2B**). Regarding the lesions with moderately differentiated there was a significant difference in the trajectory of the incidence density favoring the SRL group (p = 0.001, **Figure 2C**). The trajectory of the incidence density of poorly differentiated lesions was similar between the two groups (**Figure 2D**).

#### Safety

Kidney function remained stable in both groups over the follow-up **(Table 2)**. There were no events of biopsy-proven or treated acute allograft rejection in either group. Graft loss occurred in one patient of the control group due to interstitial and tubular atrophy fibrosis. Two deaths occurred in the SRL group due to cardiovascular disease and infectious complications, respectively, and three deaths in the CNI group due to cardiovascular disease, infectious complications and metastatic cutaneous squamous cell carcinoma, respectively.

**Table 2:** Safety parameters

	SRL group n = 23	CNI group n = 19	p-value
**Kidney function (eGRF ml/min/1,73 m^2^ ± SD)**		
T0	59.3 ± 9.4	53.4 ± 20.9	0.255
6 months after T0	58.3 ± 17.6	51.4 ± 30.3	0.362
T1	56.7 ± 15.7	52.5 ± 29.5	0.558
T2	57.4 ± 26.6	52.4 ± 31.7	0.581
T3	55.3 ± 25.7	61.1 ± 35.5	0.543
**Treated acute allograft rejection, n (%)**	0	0	–
**Graft loss excluding death, n (%)**	0	1 (5)	0.283
**Deaths, n (%)**		
Cardiovascular disease	1 (4)	1 (5)	0.877
Infectious complications	1 (4)	1 (5)	0.877
Cutaneous neoplasia	0	1 (5)	0.283
**Adverse events, n (%)**		
Proteinuria (1.34g ± 0.96g)	10 (43)	–	
Thrombocytopenia (112.217/µL ± 20.407.1/µL)	9 (39)	–	
Dyslipidemia (statin use)	7 (30)	–	
Edema	13 (57)	–	

Twenty-one patients on sirolimus presented at least one adverse event: 10 had proteinuria > 0.5g, nine had thrombocytopenia, seven had hyperlipidemia and 13 had peripheral edema. None of these events required temporary or definitive discontinuation of study medication during the conversion. Four patients interrupted sirolimus because of delayed wound healing and diarrhea in the second and third years. There was no development of *de novo* donor specific antibodies after conversion to sirolimus.

## Discussion

In this prospective study, the conversion to sirolimus monotherapy in kidney transplant recipients with high-risk cSCC was associated with a significant reduction in the incidence of moderately differentiated lesions, a tendency toward a lower incidence of lesions with other features of poor prognosis, and an adequate safety and tolerability profile.

The de novo use or conversion to mTOR inhibitors have been associated with reduction in the incidence of *de novo*cSCC lesions^1–4^. Yet, long-term benefit has been limited by poor tolerability^5,6,12^ and increased risk of acute rejection when administered as monotherapy^5,12,15^. To overcome these constrains, this trial purposefully chose non-sensitized and stable kidney transplant recipients who had been diagnosed with squamous cell skin carcinoma with poor prognosis characteristics, and used conversion to monotherapy with mTOR inhibitors as adjuvant to the dermatological therapy. In this population, the potential anti-tumor benefits would outweigh the transplant-related risks.

The most noteworthy finding was a substantial decrease in the incidence density of moderately differentiated lesions (present in 83% of the patients in the sirolimus group) from the second year of sirolimus administration, compared to a significant increase in this parameter in the control group over time. This is in agreement with previous research indicating that the advantage of converting to sirolimus on the dermatologic course of SCC lesions in kidney transplant recipients requires long-term treatment. For example, in the five-year follow-up of the TUMORAPA study,^7^ survival free of new skin lesions was longer in the sirolimus-converted group over time. 

The possible mechanisms by which mTOR inhibitors act on keratinocyte carcinogenesis may explain these results, as they include sustained processes of inhibition of cell phosphorylation and proliferation, regulation of angiogenesis, reduction of cytokine release, and suppression of oncogenes such as AFT3 (activating transcription factor 3) and GRO-α (growth regulatory oncogene alpha)^8–12^. 

In this study, conversion to sirolimus was well tolerated, with no discontinuation due to adverse events in the first year after conversion. The absence of a high loading dose, unlike in previous investigations^1–3^ and in agreement with most recent reports^14–16^, and the maintenance of blood sirolimus concentrations close to 10 ng/mL were critical to achieving this outcome. Renal function was maintained, no episodes of acute allograft rejection occurred, and there was no de novo DSA within the 3-years follow-up, suggesting the effectiveness of sirolimus monotherapy in selected non-sensitized patients.

The small number of patients included, the absence of a randomized control group, and the fact that this comparator group was composed of patients with exclusion criteria or who refused conversion are some limitations of the study.

In conclusion, the stepwise conversion from CNI-based to sirolimus monotherapy may act as adjuvant to the dermatological therapy for kidney transplant recipients with high-risk cSCC, potentially improving the quality of patients’ life. The absence of an attack dose resulted in a good profile of tolerability. Finally, there was no negative impact on the mid-term results of the transplant.

## Abbreviations

– cSCC: – cutaneous squamous cell carcinoma
– mTOR: – mammalian target of rapamycin
– CNI: – calcineurin inhibitor
– SRL: – sirolimus
– AZA: – azathioprine
– GRF: – graft function rate

## Supplementary Material

O seguinte material online está disponível para o presente artigo:

**Table s1 –** Exclusion criteria for control group patients.
