# Supplementary material for: Use of sirolimus as an adjuvant therapy for kidney transplant recipients with high-risk cutaneous squamous cell carcinomas: a prospective non-randomized controlled study
Source: J Bras Nefrol. 2023 Aug 11;45(4):480–7. doi: 10.1590/2175-8239-JBN-2023-0013en (PMC10726662; doi:10.1590/2175-8239-JBN-2023-0013en)
Supplement: Supplementary file 1 [file 2175-8239-jbn-2023-0013-s1.pdf]

Braz. J. Nephrol. (São Paulo) 2023.
https://doi.org/10.1590/2175-8239-JBN-2023-0013en

Supplementary Material to "Use of sirolimus as an adjuvant therapy for kidney transplant recipients with high-risk cutaneous squamous cell carcinomas: a prospective non-randomized controlled study"

Table S1 – Exclusion criteria for control group patients.

Control Group	Exclusion Criteria
Patient 1	eGRF <40ml/min/ 1,73 m
Patient 3	post-Tx GN
Patient 5	eGRF <40ml/min/ 1,73 m
Patient 7	eGRF <40ml/min/ 1,73 m + proteinuria
Patient 14	Hepatic disorder
Patient 15	Refused conversion
Patient 17	eGRF <40ml/min/ 1,73 m
Patient 20	Refused conversion
Patient 29	Refused conversion
Patient 30	Refused conversion
Patient 35	eGRF <40ml/min/ 1,73 m
Patient 36	Refused conversion
Patient 42	Hepatic disorder
Patient 46	eGRF <40ml/min/ 1,73 m
Patient 47	Refused conversion
Patient 48	eGRF <40ml/min/ 1,73 m + retransplant
Patient 49	Refused conversion
Patient 52	PRA>50%
Patient 55	eGRF <40ml/min/ 1,73 m

eGRF: estimate glomerular filtration rate; GN: glomerulonephritis; PRA: panel reactive antibody
